# Genomic Insights of Multidrug-Resistant *Escherichia coli* From Wastewater Sources and Their Association With Clinical Pathogens in South Africa

**DOI:** 10.3389/fvets.2021.636715

**Published:** 2021-02-26

**Authors:** Joshua Mbanga, Daniel G. Amoako, Akebe L. K. Abia, Mushal Allam, Arshad Ismail, Sabiha Y. Essack

**Affiliations:** ^1^Antimicrobial Research Unit, College of Health Sciences, University of Kwazulu-Natal, Durban, South Africa; ^2^Department of Applied Biology and Biochemistry, National University of Science and Technology, Bulawayo, Zimbabwe; ^3^Sequencing Core Facility, National Institute for Communicable Diseases, National Health Laboratory Service, Johannesburg, South Africa

**Keywords:** whole-genome sequencing, antibiotic resistance, *Escherichia coli*, wastewater treatment plant, sequence types, phylogenomic analysis, public health, river water

## Abstract

There is limited information on the comparative genomic diversity of antibiotic-resistant *Escherichia coli* from wastewater. We sought to characterize environmental *E. coli* isolates belonging to various pathotypes obtained from a wastewater treatment plant (WWTP) and its receiving waters using whole-genome sequencing (WGS) and an array of bioinformatics tools to elucidate the resistomes, virulomes, mobilomes, clonality, and phylogenies. Twelve multidrug-resistant (MDR) diarrheagenic *E. coli* isolates were obtained from the final effluent of a WWTP, and the receiving river upstream and downstream of the WWTP were sequenced on an Illumina MiSeq machine. The multilocus sequence typing (MLST) analysis revealed that multiple sequence types (STs), the most common of which was ST69 (*n* = 4) and ST10 (*n* = 2), followed by singletons belonging to ST372, ST101, ST569, ST218, and ST200. One isolate was assigned to a novel ST ST11351. A total of 66.7% isolates were positive for β-lactamase genes with 58.3% harboring the *bla*_TEM1B_ gene and a single isolate the bla_CTX−M−14_ and bla_CTX−M−55_ extended-spectrum β-lactamase (ESBL) genes. One isolate was positive for the *mcr*-9 mobilized colistin resistance gene. Most antibiotic resistance genes (ARGs) were associated with mobile genetic support: class 1 integrons (In22, In54, In191, and In369), insertion sequences (ISs), and/or transposons (Tn402 or Tn21). A total of 31 virulence genes were identified across the study isolates, including those responsible for adhesion (*lpfA, iha*, and *aggR*), immunity (*air, gad*, and *iss*), and toxins (*senB, vat, astA*, and *sat*). The virulence genes were mostly associated with IS (IS1, IS3, IS91, IS66, IS630, and IS481) or prophages. Co-resistance to heavy metal/biocide, antibiotics were evident in several isolates. The phylogenomic analysis with South African *E. coli* isolates from different sources (animals, birds, and humans) revealed that isolates from this study mostly clustered with clinical isolates. Phylogenetics linked with metadata revealed that isolates did not cluster according to source but according to ST. The occurrence of pathogenic and MDR isolates in the WWTP effluent and the associated river is a public health concern.

## Introduction

The role of the environment in the spread of antibiotic resistance is an evolving issue (1). Wastewater treatment plants (WWTPs) have received a lot of attention because of the central role they play in reducing pollutant loads that include antibiotic-resistant bacteria (ARB), antibiotic resistance genes (ARGs), virulence genes, and their associated mobile genetic elements to acceptable limits before the discharge of treated effluent into receiving water bodies.

With inadequately maintained sanitation infrastructure, low- and middle-income countries (LMICs) and emerging economies like South Africa face challenges with the release of untreated or poorly treated effluent into the environment, which may be a driver for the dissemination of antibiotic resistance in these settings (2). Constant monitoring of WWTPs for the release of multi-drug resistant (MDR) bacteria into receiving waters *via* their effluents is important as it indicates what is disseminated to the environment.

The WWTP investigated in this study is the largest in Pietermaritzburg, the provincial capital of KwaZulu-Natal in South Africa. Runoff from this WWTP is released into the Msunduzi River, a tributary that ultimately discharges into the Umgeni River (3). Upstream of the WWTP, the Msunduzi River receives runoff from rural communities, agricultural areas, urban municipalities (including several hospitals and community health centers), and numerous informal settlements along the river (4). The surface water is a key water source for domestic, agricultural, and recreational purposes to inhabitants of the several informal settlements along its banks. The river water has previously been considered to be polluted with fecal matter and unsuitable for anthropogenic activities (4).

Diarrheagenic *Escherichia coli* pathotypes are a public health concern (5). Pathogenic MDR *E. coli* that affects humans and animals have been reported in the water environment (6–8). However, studies that employ sequencing technologies to investigate environmental *E. coli* or any other bacteria are rare in Africa, including in South Africa. Consequently, there is little information regarding environmental isolates and their association with other isolates from clinical and agricultural sources. We sought to compare the genomics of MDR environmental *E. coli* isolates belonging to various pathotypes obtained from a WWTP and its receiving waters using whole-genome sequencing (WGS) and bioinformatics tools in terms of their lineages, resistomes, virulomes, mobilomes, clonality, and phylogenies to determine associations/correlations with clinical, animal, and environmental isolates.

## Materials and Methods

### Ethical Consideration

Ethical approval was received from the Biomedical Research Ethics Committee (Reference: BCA444/16) of the University of KwaZulu-Natal. Permission to collect water samples was sought and granted by Umgeni Water, which owns and operates the investigated WWTP.

### Study Site and Sample Description

A longitudinal antibiotic resistance surveillance study was undertaken in the uMgungundlovu District, one of 11 districts in the coastal province of KwaZulu-Natal, South Africa. Water samples were collected fortnightly for 7 months from May to November 2018 at the largest urban WWTP in the district. Manual grab water samples were collected in sterile 500-ml containers according to Kalkhajeh et al. (9), upstream (29°36′10.73″S and 30°25′29.97″E), downstream (29°36′27.54″S 30°27′0.76″E), and from the influent (29°36′3.70″S 30°25′41.71″E) and final effluent (29°35′49.97″S 30°26′19.74″E) of the WWTP.

### Bacterial Identification

A total of 580 *E. coli* isolates were putatively identified during enumeration using the Colilert^®^-18 Quanti-Tray^®^ 2000 system, followed by phenotypic confirmation on eosin methylene blue (EMB) agar. Briefly, before analysis, bottles containing the water samples were thoroughly mixed and then serially diluted using 10-fold dilutions. Samples from upstream and downstream river water as well as final effluent were diluted 1 ml in 100 ml (0.01 dilution) using sterile water. The influent samples were also diluted by 0.05 ml in 100 ml (0.0005 dilution) using sterile water. The 100 ml from each sample was then analyzed using the Colilert^®^-18 Quanti-Tray^®^ 2000 System (IDEXX Laboratories (Pty) Ltd., Johannesburg, South Africa). *E. coli* was obtained from positive Quanti-Trays, subcultured on EMB (Merck, Darmstadt, Germany) and incubated at 37°C for 18–24 h. At least 10 distinct colonies representing each sampling site were randomly selected from the EMB and further subcultured onto the same medium to obtain pure colonies. Molecular confirmation of the selected *E. coli* isolates was accomplished using real-time PCR targeting the *uidA* (β-D-glucuronidase) gene, as was the delineation of *E. coli* into various diarrheagenic pathotypes [i.e., enterohemorrhagic *E. coli* (EHEC), enteropathogenic *E. coli* (EPEC), enteroaggregative *E. coli* (EAEC), enterotoxigenic *E. coli* (ETEC), and enteroinvasive *E. coli* (EIEC)]. All reactions included a no-template control consisting of the reaction mixture. The real-time PCR protocol was done according to Mbanga et al. (10). The primers, virulence genes, and reference strains used to determine pathotypes are shown in Supplementary Table 1. The WGS study sample consisted of a subset of 12 MDR diarrhoeagenic isolates obtained from the upstream, downstream, and effluent sites over the study period. The selection of isolates was based on their antibiograms and pathotypes.

### Antimicrobial Susceptibility Testing

The resistance of *E. coli* to a panel of 20 antibiotics was determined through the disk diffusion assay, and the results were interpreted according to the European Committee on Antimicrobial Susceptibility Testing (EUCAST) criteria (11). The panel consisted of amikacin (AMK, 30 μg), ampicillin (AMP, 10 μg), azithromycin (AZM, 15 μg), amoxicillin–clavulanic acid (AMC, 30 μg), cefepime (FEP, 10 μg), cefotaxime (CTX, 30 μg), cefoxitin (FOX, 30 μg), ceftazidime (CAZ, 30 μg), ceftriaxone (CRO, 30 μg), cephalexin (LEX, 30 μg), ciprofloxacin (CIP, 5 μg), chloramphenicol (CHL, 30 μg), gentamicin (GEN, 10 μg), imipenem (IPM, 10 μg), meropenem (MEM, 10 μg), nalidixic acid (NAL, 30 μg), piperacillin–tazobactam (TZP, 110 μg), tetracycline (TET, 30 μg), tigecycline (TGC, 15 μg), and trimethoprim–sulfamethoxazole (SXT, 25 μg) (Oxoid Ltd., Basingstoke, UK). Breakpoints for AZM, TET, and NAL were obtained from the Clinical and Laboratory Standards Institute (CLSI) interpretative charts (12). Colistin resistance was undertaken using the broth microdilution assay to determine the colistin minimum inhibitory concentration (MIC). The results were interpreted according to the EUCAST guidelines (11). *E. coli* ATCC 25922 was used as the control.

### Whole-Genome Sequencing and Bioinformatic Analysis

The genomic DNA was extracted from the *E. coli* isolates using the GenElute Bacterial Genomic DNA Kit (Sigma Aldrich, St. Louis, MO, USA) following the instructions of the manufacturer before quantification using the 260/280 nm wavelength on a Nanodrop 8000 (Thermo Fisher Scientific Waltham, MA, USA). Library preparation was done using the Nextera XT DNA Library Preparation Kit (Illumina, San Diego, CA, USA) followed by WGS using an Illumina MiSeq Machine (Illumina, USA). Quality trimming of raw reads was done using Sickle v1.33 (https://github.com/najoshi/sickle). The raw reads were then assembled spontaneously using the SPAdes v3.6.2 assembler (https://cab.spbu.ru/software/spades/). All contiguous sequences were subsequently submitted to GenBank and assigned accession numbers (Supplementary Table 2) under BioProject PRJNA609073.

The assembled genomes were analyzed for multilocus sequence typing (MLST) sequence types (STs) on the MLST 1.8 database hosted by the Center for Genomic Epidemiology (CGE) (http://cge.cbs.dtu.dk/services/MLST/). Isolates without STs were submitted to the EnteroBase *Escherichia*/*Shigella* database (https://enterobase.warwick.ac.uk/species/index/ecoli) and assigned novel STs.

Mutations conferring resistance to fluoroquinolones were determined from the assembled genomes using BLASTN (https://blast.ncbi.nlm.nih.gov/Blast.cgi?PAGE_TYPE=BlastSearch). Briefly, DNA gyrase (*gyrA* and *gyrB*) and DNA topoisomerase IV (*par*C and *parE*) genes and the reference strain *E. coli* ATCC 25922 (Accession number: CP009072) were aligned with the genomes of this study using BLASTN. The mutations in the isolate of the genomes of this study were manually curated and tabulated.

Plasmid replicons types were identified using PlasmidFinder 2.1 on the CGE website (https://cge.cbs.dtu.dk/services/PlasmidFinder/). The assembled genomes were further analyzed for mobile genetic elements (MGEs), including insertion sequences using ISFinder (https://isfinder.biotoul.fr/) and intact prophages using PHASTER (https://phaster.ca/). RAST SEEDVIEWER (https://rast.nmpdr.org/seedviewer.cgi) was also used to annotate and identify the investigated genomes for integrons. Virulence genes were assayed using VirulenceFinder 2.0 on the CGE website (https://cge.cbs.dtu.dk/services/VirulenceFinder/). The synteny and genetic environment of ARGs and associated MGEs was investigated using the general feature format (GFF3) files from GenBank. The genetic environment of virulence genes detected in the study was also determined using a similar approach. The GFF files were imported into Geneious Prime 2020.2 (https://www.geneious.com) for analysis.

### Phylogenomic Analyses of the *E. coli* Isolates (*n* = 12) and Isolates From South Africa

Whole-genome sequences of all isolates were uploaded and analyzed on the CSI Phylogeny 1.4 pipeline (https://cge.cbs.dtu.dk/services/CSIPhylogeny/). CSI Phylogeny recognizes, screens, and validates the location of single-nucleotide polymorphisms (SNPs), before deducing a phylogeny founded on the concatenated alignment of the high-quality SNPs. Selection of SNPs was based on default parameters on the CSI Phylogeny, which included: a minimum distance of 10 bp between each SNP, a minimum of 10% of the average depth, mapping quality was above 25, SNP quality above 30, and all insertions and deletions (INDELs) were excluded. The *Morganella morganii* subsp. *morganii* KT genome (Accession number: CP004345.1) served as the outgroup to root the tree enabling the easy configuration of the phylogenetic distance between the isolates on the branches. The phylogeny was visualized with annotations for isolate information and *in-silico* typing (ST) metadata using Phandango (https://jameshadfield.github.io/phandango/#/main) to provide insights into the generated tree.

Additionally, WGS of *E. coli* isolates from South Africa curated at the PATRIC website (https://www.patricbrc.org/) were downloaded and used alongside the isolates of this study for the whole-genome phylogeny analysis to ensure a current epidemiological and evolutionary analysis (Dataset 1). The generated phylogenetic trees were visualized, annotated, and edited using iTOL (https://itol.embl.de/) and Figtree (http://tree.bio.ed.ac.uk/software/figtree/). Isolates of the same host (human, animal, or bird) or from the environment were highlighted with the same color.

## Results

### Isolate Characteristics

The 12 *E. coli* isolates investigated in this study were obtained from the WWTP and its associated waters. Seven isolates were from the downstream site, four were from the upstream site, and one isolate was obtained from the final effluent. The isolates belonged to the diarrheagenic group of *E. coli*; seven were EAEC, three were EIEC, with one EHEC, and one EPEC (Supplementary Table 2).

### Antibiotic Susceptibility

The 12 isolates had varying phenotypic resistance patterns, with most being resistant to AMP (83.3%), SXT (75%), and TET (66.7%) (Table 1). Some isolates had the same resistance profiles but were isolated at different times (months) from different sampling points. The resistance profiles AMP—TET–NAL–SXT, TET–NAL–CIP–SXT, and FOX–AMP–AMC–TET–LEX were common to isolates obtained from the downstream and upstream sites of the WWTP. Three isolates, one from downstream and one from the final effluent, had the resistance profile AMP–AMC–SXT (Table 1). The remaining four isolates had unique resistance profiles AMP–TET–AZM–SXT, AMP–TET–SXT, FOX–AMP–AMC, and AMP–AMC–LEX–CTX–CAZ–CRO–FEP–SXT but were all resistant to AMP.

**Table 1:** Source, sequence types (STs), antibiograms, resistance genes, virulence genes, and mobile genetic elements found in the *Escherichia coli* isolates.

**Isolate 1D**	**MLST**	**Source**	**Antibiogram**	**ESBL**	**ARGs**	**Integrons**	**Cassette arrays**				**Transposons**	**Virulence genes**	**Plasmid replicons**
							**GC1**	**GC2**	**GC3**	**GC4**			
D18/5	ST372	Downstream	AMP-TET-NAL-SXT	+	*blaTEM-1B, dfrA7, sul1, sul2, tet(A), mdf(A), aph(3”)-Ib, aph(6)-Id*	*In22*	*sul1*	*qacEΔ1*	*dfrA7*	*-*	-	*vat, gad, iss*	IncFIA, IncFIB IncFII (pRSB107), IncQ1
D47/7	ST69	Downstream	AMP-TET-AZM-SXT	+	*blaTEM-1B, dfrA1, dfrA14, dfrA17, sul1, sul2, tet(A), mdf(A), mph(A), aadA1, aadA5, aph (3”)-Ib, aph (6)-Id, qnrB19, qnrS*	*In54 In191 In369*	*sul1 dfrA14 aadA1*	*qacEΔ1 - dfrA1*	*aadA5 - -*	*dfrA17 - ‘-*	-	*eilA,lpfA,nfaE, air,gad,iss*,	Col156, Col440I, ColpVC, IncFIB, IncFII, IncFII(pCoo)
D64/7	ST101	Downstream	AMP-TET-SXT	-	*tet(A), tet(M), mdf(A)*	*In0*	*-*	*-*	*-*	*-*	Tn402 (Tn5090)	*lpfA, gad, iss*	IncFII
D69/7	ST218	Downstream	AMP-AMC-SXT	-	*mdf(A)*	*-*	*-*	*-*	*-*	*-*	-	*pet, aap, aatA, iha, iss, mchB, mchC, mchF, capU*	IncFII, IncFII(pCoo)
D77/8	ST200	Downstream	TET-NAL-CIP-SXT	+	*blaTEM-1B, dfrA7, sul1, sul2, mdf(A), aph(3”)-Ib,aph(6)-Id*	*In22*	*sul1*	*qacEΔ1*	*dfrA7*	*-*	Tn21	*astA, pet, pic, sat, aafA, aafB, aafC, aafD, aap, aar, aatA, aggR, iha, lpfA, gad, mchB, mchC, mchF, ORF3, ORF4, aaiC, capU*	IncFIC(FII), IncQ1
D96/9	ST69	Downstream	FOX-AMP-AMC-TET-LEX	+	*blaTEM-1B, tet(A), mdf(A), mcr-9*	*-*	*-*	*-*	*-*	*-*	-	*eilA, lpfA, tsh, iroN, gad, iss, mchB, mchC, mchF, mcmA*	IncFIA, IncFIB IncFIC(FII)
D106/9	ST11351	Downstream	AMP-AMC-LEX-CTX-CAZ -CRO-FEP-SXT	+	*blaCTX-M-14, blaCTX-M-55, dfrA14, sul2, mdf(A), aph(3”)-Ib, aph(6)-Id*	*-*	*-*	*-*	*-*	*-*	-	*eilA, lpfA, air, gad*,	IncI1, IncY
E13/5	ST569	Effluent	AMP-AMC-SXT	+	*blaTEM-1B, dfrA17, sul1, sul2, mdf(A), mph(A), aadA5, aph(3”)-Ib, aph(6)-Id*	*In54*	*sul1*	*qacEΔ1*	*aadA5*	*dfrA17*	Tn21	*senB, vat, gad, iss*	Col156, IncFIB, IncFII(29, IncQ1
U40/6	ST69	Upstream	FOX-AMP-AMC-TET-LEX	+	*blaTEM-1B, dfrA7, sul1, sul2, tet (A), mdf(A), aadA5, aph(3”)-Ib, aph(6)-Id*	*In22*	*sul1*	*qacEΔ1*	*dfrA7*	*-*	Tn402 (Tn5090), Tn21	*astA, eilA, lpfA, air, gad, iss*	Col440I, IncFII(pCoo), IncQ1, IncX4
U69/7	ST10	Upstream	TET-NAL-CIP-SXT	-	*dfrA14, sul2, tet(A), mdf(A)*	*1n191*	*dfrA14*	*-*	*-*	*-*	-	*gad*	IncFIB(pKPHS1), IncFII(K), IncI1, IncR, IncX2, IncX4
U88/8	ST10	Upstream	FOX-AMP-AMC	-	*mdf(A)*	*-*	*-*	*-*	*-*	*-*	-	*gad*	No plasmids
U117/10	ST69	Upstream	AMP-TET-NAL-SXT	+	*blaTEM-1B, dfrA14, sul2, tet(B), mdf(A), mph(A), aph(3”)-Ib,aph(6)-Id*	*1n191*	*dfrA14*	*-*	*-*	*-*	Tn402 (Tn5090)	*senB, eilA, lpfA, air, gad, iss*	Col156, IncFIA, IncFIB, IncFII(pRSB107)

*AMP, ampicillin; AZM, azithromycin; AMC, amoxicillin-clavulanic acid; FOX, cefoxitin; LEX, cephalexin; CIP, ciprofloxacin; NAL, nalidixic acid; TET, tetracycline; SXT, trimethoprim-sulfamethoxazole; bold, Novel type; ARGs, antibiotic resistance genes; ESBL, Extended spectrum β-lactamases; MLST, Multilocus sequence type; + /-, Presence/Absence*.

### Genome Characteristics

The genomic characteristics of the *E. coli* sequences are presented in Supplementary Table 2. The total assembled genome size ranged from 4.7 to 6.1 MB; the GC content ranged from 50.4 to 51.2; and the N50, L50, and the total number of contigs are also shown in Supplementary Table 2.

### Antibiotic Resistance Genes

All the *E. coli* isolates harbored ARGs, which included the β-lactamases. A total of 8/12 (66.7%) isolates were positive for the β-lactamase genes, with 7 (58.3%) harboring the *bla*_TEM1B_ gene (Table 1). One novel isolate, D106, was ESBL positive for the *bla*_*CTX*__−M−14_ and *bla*_*CTX*__−M−55_ genes but harbored different genes from other ESBL positive isolates. All ST69 isolates were ESBL positive (*bla*_TEM1B_ gene), whereas the ST10 isolates were ESBL negative. Additional genes included the *aph*(3″')-Ib, *aph*(6)-Id, *aad*A1, and *aad*A5 (which confer resistance to aminoglycosides); *tet*(*A), tet(M)*, and *tet(B)* (resistance to tetracycline), *sul1* and *sul2* (resistance to sulfonamides), *dfr*A1, *dfr*A14, and *dfr*A17 (resistance to trimethoprim), and *mdf* (A) and *mph*(A) (which confer resistance to macrolides). Notably, one isolate (D96) was positive for the *mcr*-9 gene, which confers resistance to colistin, and another (D47) was positive for the *qnr*B19 gene, which has been implicated in plasmid-mediated quinolone resistance. Tetracycline resistance determinants *tet(M)* and *tet(A)* occurred together in an EPEC isolate (D64), which was phenotypically resistant to tetracycline, with no zone of inhibition (Table 1).

The quinolone resistance determinant regions (QRDRs) were investigated for mutations in all isolates. The QRDR consists of DNA gyrase (*gyr*A and *gyr*B) and DNA topoisomerase IV (*par*C and *par*E) genes. The *gyr*A gene (S83L, D678E, L447M, A828S, and D87N), the *gyr*B gene (E703D, A618T, E185D, E58D, and I60V), and the *par*C gene (E62K, D475E, M241I, T718A, and S80I) all had five mutations while the *par*E had four mutations (V136I^*^, K146T, D475E, and L416F) (Table 2). Two isolates, E13 and U69, had mutations in all four QRDR genes. Isolate U69 had known mutations *gyrA* (S83L and D87N) that confers resistance to NAL and CIP, and *parC* (S80I) and *parE* (L416F) that confer resistance to CIP. Isolate E13, which contained unique mutations *gyrA* (D678E^*^ and A828S^*^), *gyrB* (A618T^*^), *parC* (E62K^*^, D475E^*^, and T718A^*^), and *parE* (V136I^*^, K146T^*^, and D475E^*^) was susceptible to all investigated quinolones.

**Table 2:** Point mutation table for gyrA/B and parC/E genes of environmental *E. coli*.

***Isolate ID***	**MLST**	***gyr A***	***gyr B***	***parC***	***parE***
D18/5	ST372	S83L	-	E62K^*^, D475E^*^	V136I^*^
D47/7	ST69	D678E^*^	E703D^*^	E62K^*^	-
D64/7	ST101	-	-	E62K^*^	-
D69/7	ST218	-	-	E62K^*^, M241I^*^	-
D77/8	ST200	L447M^*^	-	E62K^*^	-
D96/9	ST69	D678E^*^	E703D^*^	E62K^*^	-
D106/9	**ST11351**	D678E^*^	-	E62K^*^	-
E13/5	ST569	D678E^*^, A828S^*^	A618T^*^	E62K^*^, D475E^*^, T718A^*^	V136I^*^, K146T^*^, D475E^*^
U40/6	ST69	D678E^*^,	E703D^*^	E62K^*^	-
U69/7	ST10	S83L, D87N	E185D^*^	S80I, E62K^*^	L416F
U88/8	ST10	-	E58D^*^, I60V^*^	E62K^*^	-
U117/10	ST69	S83L, D678E^*^	E703D^*^	S80I, E62K^*^	-

* *Putatively novel mutations: novel sequence type (bold)*.

### Mobilome (Plasmids, Insertion Sequences, Intact Prophages, and Integrons)

The IncFII was the most detected plasmid replicon, with 10 (83.3%) isolates harboring it (Table 1). The IncFIA (25%), IncFIB (50%), IncQ1 (33.3%), IncI1 (16.7%), Col156 (25%), and Col440I (16.7%) plasmid replicons were also detected in some isolates. Most isolates (83.3%) had more than one plasmid replicon; however, no plasmids were detected in one isolate, U88 (Table 1). Of note, there was no unique pattern with respect to the replicon type, ST, and source of isolation.

Class 1 integrons were identified in 8 (66.7%) of the isolates of which 5 had the *qacE*Δ*1, sul1* genes, which are typically found at the 3'conserved segment in a class 1 integron (Table 1). The resistance gene cassettes identified in this study mostly harbored genes encoding resistance to trimethoprim (*n* = 8), streptomycin/spectinomycin (*n* = 2), and aminoglycosides (*n* = 2). The most frequently identified gene cassettes were *dfrA7* and *dfrA1*. Identified integron types included In22, In54, In191, and In369. Isolate D47 had three integron types: In54, In191, and In369 (Table 1). Similar integron types with identical gene cassettes were identified in isolates from different clonal types and sampling sites. Isolates from the upstream U69 (ST10), U117 (ST69), and downstream D47 (ST69) had the In191 integron type with identical gene cassette (*dfrA14* gene). Isolates from the upstream U40 (ST69) and downstream D77 (ST200) sites had the In22 with identical gene cassettes sul1, *qacE*Δ*1*, and *dfrA7*. Cassette arrays did not follow clonal lineages or source (upstream, downstream, and effluent), while isolates belonging to the same STs had different gene cassettes (Table 1). Some of the class 1 integrons were bracketed by transposons [e.g., U117 (ST69)]. The integron was flanked by the *TniA* and *TniB* genes associated with the Tn402-like transposons. Two other isolates, D77 (ST200) and E13 (ST569), had class 1 integrons flanked by the Tn21 transposon, which belongs to the Tn3 group of transposons. A transposable element Tn402 (Tn5090) not linked to an integron was identified in D64 (ST101). The class 1 integron in U40 (ST69) was flanked on one side by Tn21, with the other end harboring the *TniA* and *TniB* genes (Table 1). The fluidity and mobility of MGEs was evident from the different permutations and combinations in isolates from different sources (Table 1).

The ARGs were mostly co-carried on class 1 integrons or associated with insertion sequences and/or transposons (Table 3). The *bla*_TEM−1B_ gene was commonly associated with a recombinase, and the IS91 insertion sequence was the most common insertion sequence. The IS91 was also associated with aminoglycoside, trimethoprim, and sulfonamide resistance genes. Insertion sequences, IS5 and IS6, were also found associated with the *bla*_CTX−M55_ and *mcr-9* genes, respectively. Tn3 transposons occurred either independently or with class 1 integrons (Table 3). The resistance genes and MGEs in the *E. coli* isolates were closely related (98–100% similarity) with target sequences in the GenBank database. Most hits were for plasmids, with the most common being the *E. coli* EcPF40 plasmid p1 (CP054215.1). The rich diversity of ISs and transposons attests to the plasticity of the bacterial genomes and horizontal gene transfer (HGT) of ARGs within and between different isolates.

**Table 3:** Mobile genetic elements associated with antibiotic resistance genes in environmental *E. coli*.

**Isolate (MLST)**	**Contig**	**Synteny of resistance genes and MGE**	**Plasmid/chromosomal sequence with closest nucleotide homology (accession number)**
D18/5 (ST372)	7	IS91: *bla*_TEM−1B_: recombinase	*E. coli* EcP40 plasmid p1 (CP054215.1)
	21	Transposase:*tetR*(A):*tet(A)*:	*E. coli* AR_0013 chromosome (CP032204.1)
	52	*sul*1: qacEΔ1: *dfrA*7:*Intl1*	*E. coli* SCU_164 chromosome (CP054343.1)
	84	*aph(6)-Id*: *aph(3”)-Ib*: *sul2*	*K. pneumoniae* plasmid p19051-FIIK (MN823997.1)
	21^*^	*merE:merD:mer(II)reductase:merC:merP:merT:merR:transposase:tetR(A):tet(A)*	*E. coli* AR_0013 Chromosome (CP032204.1)
D47/7(ST69)	2	*pspF*: *QnrB19*:	*K. pneumoniae* plasmid pRIVM_C014947_7 (MT560070.1)
	78^*^	*padR:chrA::sul1*: qacEΔ1:ant*(3”)-Ia:dfrA17: Intl1*	*E. coli* strain AH62 plasmid pAH62-3 (CP055262.1)
	95	*bla*_TEM−1B_	*K. quasipneumoniae* strain S174-1 plasmid pS174-1.1(CP063875.1)
	201	*tetR*-FTR (AcrR):: *mph(A)*	*K. pneumoniae* strain S90-2 plasmid pS90-2.3 (CP063884.1)
	390	*aadA1*: *dfrA1*	*E. coli* strain EcPF5 plasmid p1 (CP054237.1)
	401	*dfrA14*	*K. pneumoniae* strain KP20194a plasmid pKP20194a-p3 (CP054783.1)
	430	*tet(A)*: *tetR*(A):relaxase: *aph(6)-Id: aph(3”)-Ib:sul2*	*E. coli* strain SCU-103 plasmid pSCU-103-1 (CP054458.1)
D64/7(ST101)	32	*tet(A)*: *tetR*(A):transposase	*E. coli* strain CFS3292 plasmid pCFS3292-1 (CP026936.2)
	36	*tetrLpep: tet(M):*	*E. coli* strain CFS3292 plasmid pCFS3292-1 (CP026936.2)
D77/8(ST200)	2	*sul1*:qacEΔ1: *dfrA*7: *IntI1::recombinase:Tn3(TnAs3)::ISI*	*E. coli* strain RHBSTW-00014 chromosome (CP056902.1)
	62	IS91: *bla*_TEM−1B_:recombinase	*E. coli* EcPF40 plasmid p1 (CP054215.1)
	67	*aph(6)-Id: aph(3”)-Ib: sul2:*	*E. coli* F070 plasmid pF070-NDM5 (AP023238.1)
D96/9(ST69)	91	*repA* (incFII):Tn3:recombinase: *bla*_TEM−1B_::recombinase: Tn3 (TnAs1)	*E. coli* strain EC28 plasmid p2 (CP049102.1)
	131	*tet(A)*: *tetR*(A):transposase	*E. coli* strain CFS3273 plasmid pCFS3273-1 (CP026933.2)
	190	*mcr-9*: *WbuC*: IS6 (IS26)	*E. coli* CVM N18EC0432 plasmid pN18EC0432-1 (CP048293.1)
D106/9 (ST11351)	2	*sul2::::IS91: aph(6)-Id: aph(3”)-Ib: dfrA14*	*Citrobacter freundii* plasmid pRHB16-C09_5 (CP057750.1)
	32	IS5: *bla_*CTX*−*M*−14_*	*K. pneumoniae* plasmid pB16KP0177-4 (CP052528.1)
	42	*bla_*CTX*−*M*−55_: WbuC::: ParA*	*E. coli* strain RD174 plasmid pHNRD174 (KX246268.1)
E13/5(ST569)	28^*^	*PadR:chrA::sul1*: *qacEΔ1*:aadA5*:dfrA17: Intl1::*recombinase	*E. coli* strain SCU-482 plasmid pSCU-482-1 (CP040859.1)
	31	*aph(6)-Id: aph(3”)-Ib: sul2:*	*E. coli* strain EcPF40 plasmid p1 (CP054215.1)
	34	DDE integrase: *tetR*-FTR (AcrR)::: m*ph(A)*	*K. pneumoniae* strain S183-1 plasmid pS183-1.2 (CP063929.1)
	36	IS91:*bla*_TEM−1B_:recombinase	*E. coli* strain EcPF40 plasmid p1 (CP054215.1)
U40/6(ST69)	39	*transposase:tetR(A):tet(A)*	*E. coli* strain CFS3273 plasmid pCFS3273-1 (CP026933.2)
	40	DDE integrase:TniB::*sul1*: *qacEΔ1*:*dfrA7: Intl1::*recombinase	*E. coli* SCU_164 chromosome (CP054343.1)
	43	*aph(6)-Id: aph(3”)-Ib:sul2*	*E. coli* strain UFU_EC98 plasmid pEc98_3 (CP024095.1)
	55	IS91: *bla*_TEM−1B_	*E. coli* strain EcPF5 plasmid p1 (CP054237.1)
	39^*^	*merE:merD:mer(II)reductase:merC:merP:merT:merR:transposase:tetR(A):tet(A)*	*E. coli strain CFS3273 plasmid pCFS3273-1 (CP026933.2)*
U69/7(ST10)	3	sul2:::IS91(ISVsa3)::recombinase	*K. quasipneumoniae* plasmid pRHBSTW-00138_10 (CP058140.1)
	29	ISKra4(ISKpn19): recombinase::transposase: *tetR*(A):*tet(A)*:::Tn3 (TnAs1):recombinase	*E. coli* strain EC194 plasmid p194 (MH121703.1)
	105	DfrA14:intl1:DDE integrase	*K. pneumoniae* plasmid pF16KP0096-1 (CP052151.1)
U117/10(ST69)	54	*bla*_TEM−1B_:IS91: *aph(6)-Id: aph(3”)-Ib:sul2::IS110(IS5075):Tn3*	*K. pneumoniae* strain Xen39 plasmid unnamed1 (CP040859.1)
	59	*tetR(B):tet(B):tet(C)::IS4(ISVa5)*	*Shigella flexneri* strain FDAARGOS_714 chromosome (CP055124.1)
	66	*tetR*-FTR (AcrR):: m*ph(A)*	*K. pneumoniae* plasmid p19110124-1 (CP064175.1)
	73	*mobC*:*dfrA14*:*intl1*	*E. coli* strain DH5alpha plasmid pESBL162 (MT230135.1)

* *Co-occurrence of a heavy metal resistance gene (HMRG), disinfectant resistance gene (DRG), and antibiotic resistance genes (ARGs)*.

The co-carriage of heavy metal (mercury and chromate), disinfectant (quaternary ammonium compounds), and ARGs were evident in several isolates. The mercury resistance operon was found associated with a transposase, tetracycline resistance transcriptional repressor *tetR(A*), and the tetracycline resistance gene *tet(A*) in two isolates from the upstream (U40) and downstream (D16) sites of the WWTP (Table 3). The class 1 integrons in isolates D47 (downstream site) and E13 (effluent site) had the *PadR* (a transcriptional regulator) and *chrA* (chromate transport protein) downstream and adjacent to the integrons (Table 3). The synteny of heavy metal, disinfectant, and ARGs in these isolates consisted of the *chrA* (chromate resistance)*, qacE*Δ*1* gene (which is a disinfectant resistance gene), and the class 1 integron ARG cassette (Table 3).

Phylogenetics linked with metadata revealed that isolates did not cluster according to source but according to ST (Figure 2). There was a clear association between the presence of the sulfonamide *sul2* and the aminoglycoside *aph*- genes, and the *sul1* gene and trimethoprim *dfrA* genes. The ESBL positive isolates had more resistance genes than the ESBL negative isolates; the number of resistance genes was not linked to the pathotype or clonality. Isolate D69 (ST218) was the only ESBL-negative EAEC isolate and had only one macrolide resistance gene (*mdfA*), while the other ESBL-positive EAEC isolates had more (Figure 2).

A total of 45 IS families were detected across the isolates (Supplementary Table 3). There was a great diversity of IS families, with only two occurring more than once.

A total of 19 intact prophages were found across all the investigated isolates (Supplementary Table 3). The Entero_mEp460 and Shigel_sfII were the most common prophages occurring in four different isolates each. The Entero_PsP3 (*n* = 3), Salmon_Fels_2 (*n* = 3), and Entero_fAA91_ss (*n* = 2) also occurred in several isolates. None of the prophages carried ARGs; however, some prophages carried virulence genes (Table 4). The abundance of ISs and prophages in environmental *E. coli* isolates is evidence of a very flexible genome that is constantly gaining and losing genetic elements through mobilizable regions of the genome.

**Table 4:** Mobile genetic elements associated with virulence genes in environmental *E. coli*.

**Isolate**	**Contig**	**Synteny of virulence genes and MGE**	**Plasmid/chromosomal sequence with closest nucleotide homology (accession number)**
D18 (ST372)	6	Prophagetailfibreprot::***iss***:rzoD:lysozyme:::EssD(phagelysisprotein)	*E. coli* CFTO73 chromosome (AE014075.1)
	73	Integrase:IS1:***vat***	*E.coli* strain 144 chromosome (CP041550.1)
D47/7(ST69)	38	IS66transposase:IS66:IS66:***nfaE***	*E. coli* strain 118UI chromosome (CP032515.1)
	144	***Iss***:rzoD:RrrD(lysozyme):phageholinprotein	*E. coli* NMBU-W13E19 chromosome (CP043406.1)
D64/7(ST101)	16	Recombinase:tailfibreassemblyprot:tailfbrpro:phageterminase:DNApackagingprot::***iss***:RzoD:lysozyme:phage lysisprot	*E. coli* strain Res13-Lact-PEB01-20 chromosome (CP062868.1)
D69/7 (ST218)	39	***mchI***:***mchB***:::***hlyD***:***mchF***	*E. coli* 2013C chromosome (CP027355.1)
	34	IS30:transposase::virK:***capU***::IS3	*E. coli* strain MEM chromosome (CP012378.1)
	39	Transposase:IS66 (TnpB):IS66:transposase:***iha***	*E. coli* 2013C chromosome (CP027355.1)
	51	DNApakagingprot::***iss***:RzoD:lysozyme(RrrD):phage lysisprot (EssD)	E. coli CFTO73 chromosome (AE014075.1)
	83	***pet***:transposase:::IS66	E. coli strain S50 plasmid (CP010239.1)
D77/8(ST200)	23	IncFII repA:transposase:***ORF3***:***ORF4***::transposase	*E. coli* RHBSTW-00014 Plasmid p RHBSTW-00014_2 (CP056903.1)
	32	***aafA***:***aafD***:IS3:IS3::IS3	*E. coli* 042plasmid pAA(FN554767.1)
	39	***aafC***:***aafB***:IS3:transposase	*E. coli* RHBSTW-00622chromosome (CP055438.1)
	45	***aaiC***:tssC:tssB:IS630:::transposase	E. coli strain 2492 chromosome (CP044021.1)
	49	***mchI***:***mchB***:::***hlyD***:***mchF***	*E. coli* 2013C chromosome (CP027355.1)
	50	IS66::IS66^*^:IS66:IS66transposase:IS630:***aar***:IS3:IS481:***aap***:IS3:IS3:***aggR***	*E. coli* 042plasmid pAA (FN554767.1)
	59	***pic***:IS256:transposase::iucA:iucB:iucC:iucD:***iutA***::***sat***	*E. coli* strain NRRL B-1109 chromosome (CP039753.1)
	69	***Iha***:transposase:IS66^*^:IS66:ISEc22	*E. coli* strain NCTC10444 chromosome (LR134092)
D96/9(ST69)	95	***mchI***:***mchB***:***mchC***::***hlyD***:***mchF***	*E. coli* 2013C chromosome (CP027355.1)
	97	IS200.IS605::ISNCY transposase:IS3 transposase::IS3:IS3like:transposase:***iss***:lysisprot:transposase:iroB:iroC:iroD:iroE:iroN	*E. coli* strain CVM N17EC0616 plasmid pN17EC0616-1 (CP043737.1)
	121	EssD:rrrD:RzoD:***iss***	*E. coli* NMBU-W13E19 chromosome (CP043406.1)
	122	***eilA:air***	E. coli WP8-S17-ESBL-12 (AP022222.1)
	150	phageterminaseprot:DNApackprot::***iss***	*E. coli* NMBU-W13E19 chromosome (CP043406.1)
D106/9 (ST11351)	17	**eilA:air**	*E. coli* WP8-S17-ESBL-12 chromosome (AP022222.1)
E13/5(ST569)	1	Integrase:IS1:***vat***	*E. coli* strain 144 chromosome (CP041550.1)
	3	Lysozyme:RzoD:***iss***:DNApackprot:phageterminaseprot	*E. coli* NMBU-W13E19 chromosome (CP043406.1)
	12	phageHolin::RzoD:***iss***:endonuclease:phageterminase	*E. coli* strain SCU-485 chromosome (CP053245.1)
	27	***senB***:Tie:IS91	E. coli strain EcPF7 plasmid p1 (CP054233.1)
U40/6(ST69)	29	***eilA:air***	*E. coli* WP8-S17-ESBL-12 chromosome (AP022222.1)
	53	***Iss***:RzoD:RrrD	*E. coli* NMBU-W13E19 chromosome (CP043406.1)
U117/10(ST69)	39	DNApackprot:***Iss***:RzoD:RrrD:classIIholin	*E. coli* NMBU-W13E19 chromosome (CP043406.1)
	40	***eilA:air***	*E. coli* strain SCU-313 chromosome (CP051694.1)
	53	***senB***:TieB:IS91	E. coli strain EcPF7 plasmid p1 (CP054233.1)

* *Virulence gene(s) in bold*.

### Virulome and Serotypes

A total of 31 virulence genes were identified across all isolates (Table 1). Isolates obtained from downstream of the WWTP had the most virulence genes, including D77 (22 virulence genes), followed by D96 (10) and D69 (9) (Supplementary Figure 1). All isolates had at least one virulence gene, with isolates U69 and U88 (from the upstream site) having only one virulence gene each. The most common virulence genes were those encoding immunity *gad* (11 isolates), *iss* (8 isolates) and *air* (four isolates), and adhesion *Ipf* A (seven isolates) and *eil*A (five isolates) (Supplementary Figure 1).

The virulence genes were mostly associated with several insertion sequences, including IS1, IS3, IS91, IS66, IS630, and IS481, suggesting that insertion sequences play a prominent role in transferring virulence genes in environmental isolates (Table 4). The vacuolating autotransporter protein (*vat*) gene encoding a cytotoxin was mostly found with a transposase and the insertion sequence IS1. The *senB* gene, which encodes an enterotoxin, was mostly associated with IS91. The insertion sequences IS3 (*aafA, B, C, D, capU*) and IS66 (*nfaE, iha, pet*) were associated with different virulence genes. The increased serum survival (*iss*) gene was bracketed by several prophage genes, including the *RzoD* (outer membrane lipoprotein), *RrrD* (lysozyme), and *EssD* (lysis protein), implying that it is carried on a prophage (Table 4). Most of the virulence genes and their associated MGEs were similar (98–100%) to target sequences in GenBank, with the most hits being for chromosomal sequences. This indicates that *E. coli* virulence genes may mostly be carried on chromosomes.

The somatic (O) and flagellar (H) antigens were used for serotyping environmental *E. coli* isolates where nine different O antigens and 11 different H types were identified across all isolates. No O type was detected for isolate D18, which only had the H31 antigen (Supplementary Table 2). The complexity and diversity of the virulome coupled with the range of identified capsule types are worrying as they are associated with virulence. Environmental isolates have a rich repertoire of virulence genes mobilized mostly by ISs contributing to the dynamic milieu of resistance, virulence, and MGEs in the water environment.

### Sequence Types and Phylogenomic Relationships

**Figure 1:**
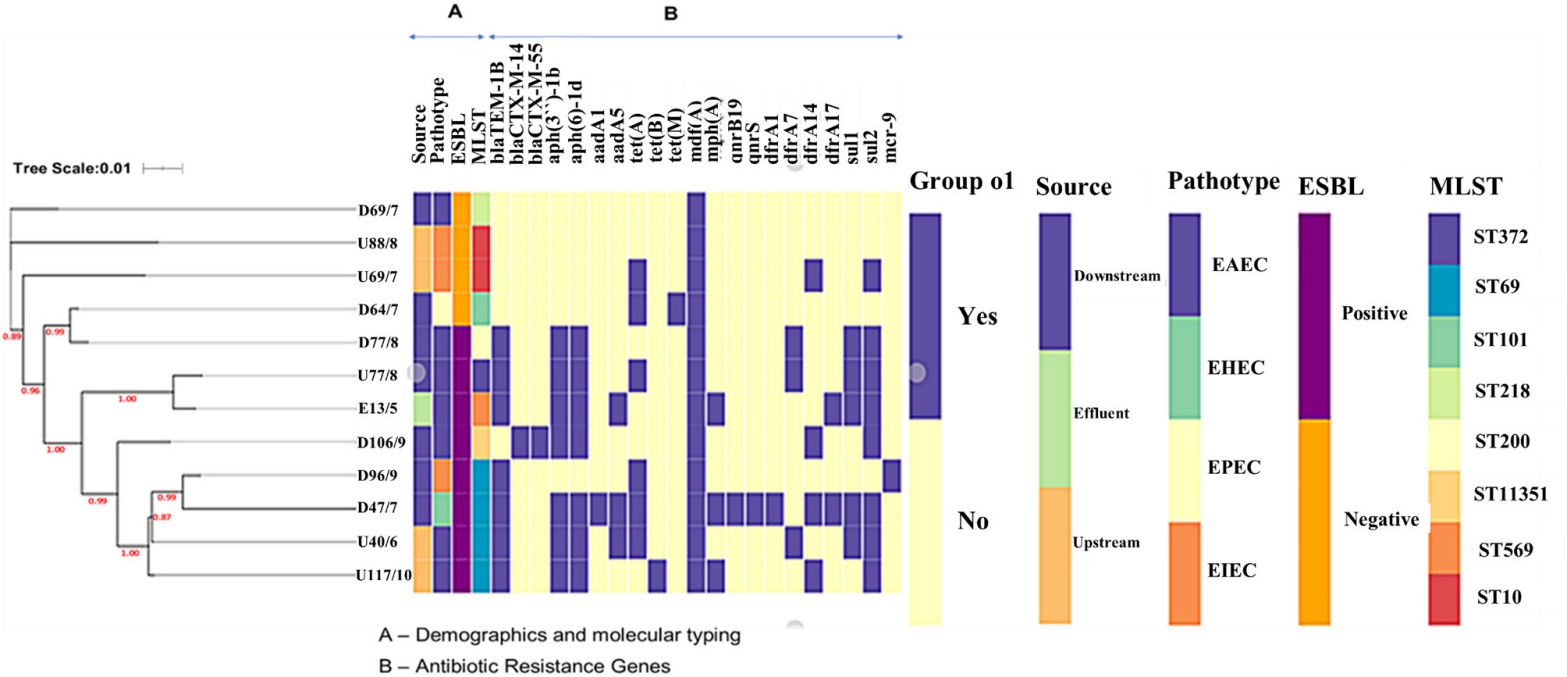
The phylogenetic branch and metadata [demographics, molecular typing, and antibiotic resistance genes (ARGs)] coupled by the use of Phandango (https://github.com/jameshadfield/phandango/wiki) in isolated multidrug resistant *Escherichia coli* strains (*n* = 12) from wastewater sources in South Africa.

The MLST analysis revealed that the *E. coli* isolates belonged to multiple STs. The most common ST was ST69 (*n* = 4), followed by ST10 (*n* = 2), the rest had unique STs ST372, ST101, ST569, ST218, and ST200 (Table 1). Isolate D106 was assigned a novel ST, ST 11351. The phylogenetic analysis combined with metadata revealed that isolates of the same MLST clustered together [e.g., isolates from downstream (D47 and D96) and upstream (U40 and U117) sites that all belonged to ST69 (Figure 1)]. However, it was interesting to note that the isolates clustered according to the isolation site, with the downstream isolates (D47 and D96) forming their subclade. Some single STs from the downstream and effluent sites also clustered together, including D18 (ST372) and E13 (ST569), and also D64 (ST101) and D77 (ST200) (Figure 1).

**Figure 2:**
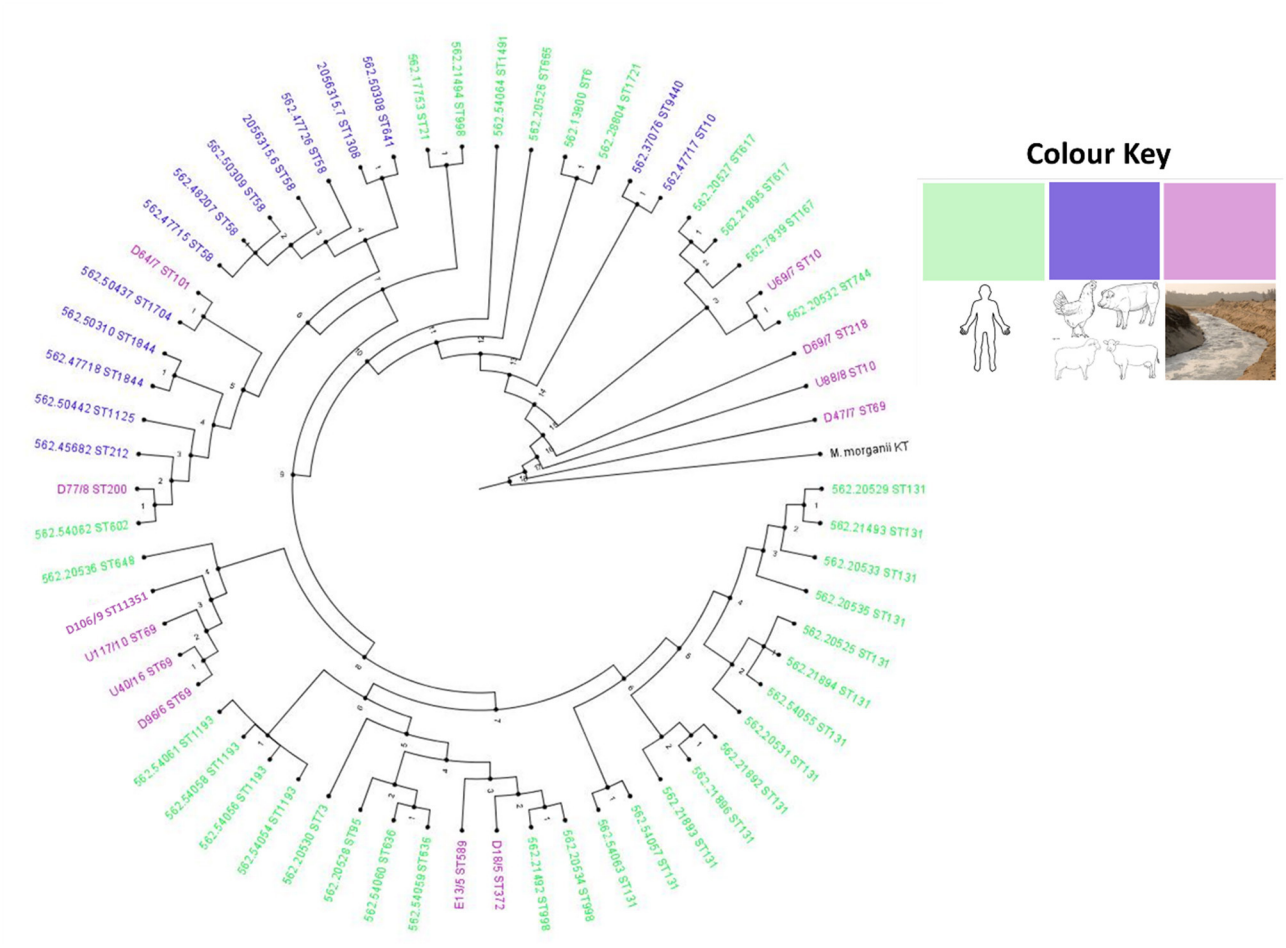
Circular phylogenomic tree with color annotations depicting the relationship between *E. coli* isolates from this study and South African isolates from diverse sources in the one-health continuum. The strains used in this study (from environmental source colored in purple) were basically related to strains from human sources (colored in green).

Compared with South African *E. coli* isolates from different sources (animals, birds, and humans), the isolates from this study mostly clustered with clinical isolates (Figure 2). Isolates U40 (ST69), D96 (ST69), U117 (ST69), and DI06 (ST11351) clustered together and were closely related to a clinical isolate (ST648) obtained from a blood sample in Pretoria Hospital. Isolates E13 (ST569) and D18 (ST372) clustered together and with other clinical isolates (ST998) obtained from urine samples from hospital patients in Pretoria. D77 (ST200) and U69 (ST10) also clustered with clinical isolates obtained from hospital patients in the Western Cape and Pretoria, respectively, albeit in different clades. An isolate D64 (ST101) was closely related to an isolate from a wild bird obtained from Durban (Figure 2). The remaining three isolates, D69 (ST218), U88 (ST10), and D47 (ST69), were more closely related to each other and did not cluster with any isolates from animals, birds, or humans and may be considered a unique aquatic lineage.

## Discussion

Genomic insights reported in this study revealed the complexity and diversity of lineages, resistome, mobilome, and virulome of MDR *E. coli* found in wastewater and river water in Kwazulu Natal, South Africa, intimating that the aquatic environment contains a fluid and dynamic milieu of ARB and ARGs. The ARGs were mostly carried on plasmids, transposable elements, and integrons, and fewer were associated with IS. The virulence genes were mostly associated with IS, which are probably central in their rearrangement and transfer. The occurrence of heavy metal, disinfectant, and ARGs in bacterial isolates is a cause for concern as it may lead to co-selection of ARB.

An assortment of ARGs and MGEs was detected among and within the sampled sites (Table 1). The variation in the ARGs and associated MGEs may reflect numerous, distinct horizontal transfer events among environmental isolates. The occurrence of ARGs, most notably the ESBL, tetracycline, sulfonamide, and macrolide genes, was not dependent on the sample source or clonal type. This contrasts with a study done in the USA that studied ESBL and *Klebsiella pneumoniae* carbapenemase (KPC) producing *E. coli* from municipal wastewater, surface water, and a WWTP. WGS of *E. coli* isolates revealed an association between the sample source and the presence of specific ESBL genes (e.g., *bla*_TEM_ was unique to municipal wastewater isolates, whereas *bla*_CTX−M_ was unique to WWTP raw influent isolates (13)). Most ARGs and associated MGEs were carried on plasmids (Table 3), signifying that plasmids play a central part in the resistome of environmental *E. coli* isolates. A few ARGs including those encoding tetracycline resistance [*tet(A), tet(B), tet(C)*], sulfonamides (*sul1*), and trimethoprim (*dfrA7*) (carried on a class 1 integron) were found on chromosomes. However, the integrons and transposons were largely associated with ARGs on plasmids, similar to findings in other studies (14). Che et al. (14) used WGS to investigate the ARGs in total DNA extracted from water samples from three WWTPs in Hong Kong and reported that ARGs carried on plasmids were dominant in the resistome of the WWTPs.

In this study, the investigated ARGs were mainly bracketed by transposons, insertion sequences, and class 1 integrons. A novel isolate, D106 (ST11351), had the *bla*_CTX−M−14_, *bla*_CTX−M−55_ genes (Table 1). Both genes were found on genetic elements IS5: *bla*_*CTX*−*M*−14_ and *bla*_*CTX*−*M*−55_*:WbuC::: ParA* on contigs that had closest nucleotide homology to plasmids from *K. pneumoniae* pB16KP0177-4 (CP052528.1) and *E. coli* pHNRD174 (KX246268.1), respectively. The isolate exhibited phenotypic resistance to tested cephalosporins, including FEP, CTX, CAZ, CRO, and LEX; this was expected since the *bla*_CTX−M−55_ is a variant of the *bla*_CTX−M−15_, which has heightened cephalosporin-hydrolyzing action (15). The *bla*_CTX−M−14_ and *bla*_CTX−M−15_ remain among the most predominant CTX-M types worldwide (16) and have also been reported in several studies on clinical isolates in South Africa (17–19). However, there are no reports on the occurrence of *bla*_CTX−M−55_ in environmental *E. coli* in South Africa. The *bla*_TEM−1B_ was found in the same genetic context *IS91: bla*_*TEM*−1*B*_*:recombinase* for isolates from the upstream (U40), effluent (E13), and downstream sites (D18 and D77) (Table 4) and had high sequence similarity to *E. coli* EcPF40 plasmid p1 (CP054215.1). The *bla*_*TEM*_genes are often plasmid mediated and are the leading cause of AMP resistance in Gram-negative bacteria (20). The IS91 can mobilize adjacent sequences through a one-ended transposition process, and the association with p1 plasmids points to a plasmid-mediated circulation of these genes in the water environment (21, 22).

An interesting finding in this study was the occurrence of the plasmid-borne *mcr-*9 gene in isolate D96 (Table 1) that also had ESBL, macrolide, and tetracycline resistance genes. Phenotypic resistance to colistin was then determined using the colistin MIC, and the isolate was found to be susceptible (<4 mg/L). The phenotypic susceptibility to colistin in isolates carrying the *mcr-9* gene was also reported in a study conducted in the USA where 100 *mcr-9* positive *Salmonella enterica* and *E. coli* isolates from the National Antimicrobial Resistance Monitoring System (NARMS), which samples retail meat, reported that all 100 isolates were susceptible to colistin, suggesting that the *mcr-9* gene may not be associated with colistin resistance (23). In this study, the *mcr-9* gene was found adjacent to an unknown function cupin fold metalloprotein gene (*WbuC*) in genetic context *mcr-9*:*WbuC*:IS6 (IS26). Similar genetic contexts have been reported in *Enterobacter hormaechei*, and *Salmonella* Typhimurium isolates from studies undertaken in the USA and China (24–26). This points to a stable *mcr-9* locus, whose transfer is mediated by insertion sequences. The *mcr-9* gene was first reported in a clinical *Salmonella* Typhimurium isolate in the USA in May 2019 (24). Isolates harboring *mcr-9* were subsequently identified in 21 countries covering six continents, including Europe, Asia, America, Oceania, South America, and Africa (27). An *mcr-9* harboring *Enterobacter hormaechei* isolate obtained from the sputum of a patient in Cairo, Egypt, is to date the only *mcr-9* positive isolate reported in Africa (28). Thus, this is the second report of the *mcr-9* positive isolate in Africa and the first in Southern Africa.

The co-occurrence of aminoglycoside resistance genes [*aph(6)-Id: aph(3”)-Ib*] with sulfonamide and trimethoprim or tetracycline genes revealed the presence of resistance islands located on regions with high similarity to plasmids deposited in GenBank. This implies that the transmission of these resistance genes is plasmid mediated (Table 3). The genetic context, *aph(6)-Id: aph(3”)-Ib:sul2*, has been found complete or incomplete within plasmids, integrative conjugative elements (ICE), and chromosomes in both Gram-negative and Gram-positive organisms (29).

Most TET-resistant isolates harbored the *tet*(A) gene and were phenotypically resistant (Table 1). Only one isolate had *tet*(B), and another isolate had *tet(A)* and *tet(M)*, and both were phenotypically resistant to TET (Table 1). In this study, the *tet(A)* gene was consistently found within a resistance operon adjacent to a transcriptional repressor gene *tetR(A)* and a transposase (Table 3). The genetic context *transposase:tetR(A):tet(A)* had high similarity to plasmid sequences deposited in GenBank, especially *E. coli* strain CFS3292 plasmid pCFS3292-1 (CP026936.2). The *tet(A)* family has been constantly associated with conjugative plasmids, which mediate transfer (30). Isolate U117 had *tet(B)* and *tet(C)* flanked by an insertion sequence IS4 (SVa5), which may be important in mobilizing these resistance genes.

The genes encoding resistance to trimethoprim/sulfonamides, *sul1, sul2*, and *dfrA* gene cassettes, were detected in 8 (66.7%) isolates. The *sul2* genes were consistently co-carried with the aminoglycoside resistance *aph-* genes [*aph(6)-Id: aph(3”)-Ib*] with *sul1* being co-carried with the *dfrA* and *qacE*Δ*1* genes (Table 1). There was concordance in the phenotypic and genotypic results in 7 (58.3%) isolates, with one isolate being phenotypically susceptible to SXT but possessing genotypic resistance traits (Table 1). A total of 8 (66.7%) isolates harbored class 1 integrons with an array of gene cassettes (Table 1). The class 1 integrons are directly linked with the Tn3 transposon family (Tn21 or Tn1697), mainly because of their inability to self-transfer; thus, they rely on conjugative plasmids and transposons for their horizontal or vertical transmission (31). Two isolates (E13 and D77) had class 1 integrons that were carried by the Tn21 transposon (Table 1). U40 was, however, unique in that either side of its class 1 integron had different transposons (namely, Tn21 and Tn402). The Tn402 (Tn5090) may carry class 1 integrons or mercury resistance integrons (*MerR*) and are characterized by *TniABQR* genes (32). The TniA codes for a putative transposase, TniB is a nucleoside triphosphate (NTP) binding protein, TniR is a resolvase or integrase, and TniQ is required for transposition (32). The class 1 integrons in isolates D47 and E13 had the *PadR* (a transcriptional regulator) and the *chrA* (chromate transport protein) downstream and adjacent to the integrons (Table 3). The *ChrA* gene is a heavy metal resistance gene (HMRG) that encodes resistance to chromate and is usually found on plasmids or chromosomes of bacteria (33). The *qacE*Δ*1* gene is a disinfectant resistance gene (DRG) that encodes resistance to disinfectants of quaternary ammonium compounds (34). A mercury resistance operon was associated with *tet(A)* resistance genes and transposons in two isolates from upstream (U40) and downstream (D18) of the WWTP (Table 3). The co-occurrence of HMRGs, DRGs, and ARGs was recently demonstrated in *E. coli* strains obtained from rivers, streams, and lakes in Brazil (8). The coexistence of HMRGs, DRGs, and ARGs in the studied integrons is important as disinfectants and heavy metals can co-select for ARGs (35). Altogether, these results revealed that ARGs carried on plasmids predominate the investigated water resistome; however, IS, transposable elements, and integrons accentuate the mobility of the plasmid-encoded ARGs and HMRGs.

A huge diversity of virulence genes often associated with pathogenic *E. coli* was found in the genomes of isolates in this study (Table 1). Similar virulence factors have been identified in environmental *E. coli* isolates obtained from surface water and WWTPs in previous studies (8, 36). Most virulence genes were associated with the insertion sequences, suggesting that these are important in the mobilization of the bacterial virulome (Table 4). The *iss* gene is responsible for increased serum survival and mediates against phagocytosis enabling the evasion of the immune system (37). The genetic environment of the *iss* gene consisted of bacteriophage genes, implying that it is carried on a prophage in *E. coli* isolates (Table 4). The *iss* gene is thought to have evolved from a λ phage gene called *bor*, which integrated into the genomes of different *E. coli* pathotypes (37). Virulence genes are frequently clustered together on the bacterial chromosome in pathogenicity islands (PAIs). In Gram-negative bacteria, the PAIs tend to contain insertion sequences that promote reorganizations and transfer of virulence genes (38). Several virulence genes, including *senB* (IS91), *vat* (IS1), and *iha* (IS66), were associated with IS (Table 4). The virulome of the environmental isolates investigated in this study revealed a diverse assemblage of virulence genes that are mobilizable and not clone specific.

Phylogenomic analyses revealed that the environmental samples in this study clustered mainly with clinical isolates, mostly from hospital patients (Figure 2). Six EAEC and two EIEC isolates were closely related to clinical isolates, implying that they originated from clinical sources. The spread of EIEC and EAEC is frequently associated with food sources or polluted water (8, 39, 40) as was the case in this study. An EPEC isolate (D64) was closely related to an isolate from a wild bird (Figure 2). Typical EPEC isolates are rarely isolated from animals as humans are the major natural reservoir; however, atypical EPEC occurs in healthy and sickly animals and humans (5). The EPEC isolate from this study probably originated from an animal host.

This study focused on a small subset of MDR and diarrheagenic *E. coli*; thus, its findings may not be generalized for all *E. coli* pathotypes. Similar studies employing a larger sample size and covering greater geographical area and diversity of *E. coli* should be conducted. However, this study adds to the knowledge that pathogenic *E. coli* can survive and be disseminated in the water environment, which is a public health concern.

## Conclusions

The occurrence of pathogenic and MDR isolates in the WWTP effluent and the associated river is a public health concern. *E. coli* isolates have a wealth of ARGs and virulence genes that have been mobilized on diverse MGEs as evident from the different permutations and combinations of ARGs, virulence genes, and MGEs in *E. coli* STs and pathotypes from the different water sources. The findings of this study may not be typical of all WWTPs and river systems in South Africa or beyond but form a basis of the need for surveillance systems that employ high-throughput technologies like WGS to gain genomic insights into the environmental dimensions of AMR. Surveillance of ARB in wastewater and associated surface waters could serve as a proxy for local antibiotic resistance and how this changes over time.

## Data Availability Statement

The datasets presented in this study can be found in online repositories. The names of the repository/repositories and accession number(s) can be found at: https://www.ncbi.nlm.nih.gov/genbank/, PRJNA609073.

## Author Contributions

SYE, ALKA, and JM co-conceptualized the study. JM, ALKA, and DGA performed the experiments. JM, ALKA, DGA, MA, and AI analyzed the data. JM wrote the paper. SYE, ALKA, and DGA supervised. SYE involved in funding acquisition. All the authors undertook critical revision of the manuscript and also reviewed, edited, and approved the final manuscript.

## Conflict of Interest

SYE is chairperson of the Global Respiratory Infection Partnership and member of the Global Hygiene Council, both funded by unrestricted educational grants from Reckitt and Benckiser (Pty.), UK. The remaining authors declare that the research was conducted in the absence of any commercial or financial relationships that could be construed as a potential conflict of interest.
