# Supplementary material for: Genomic Insights of Multidrug-Resistant Escherichia coli From Wastewater Sources and Their Association With Clinical Pathogens in South Africa
Source: Front Vet Sci. 2021 Feb 26;8:636715. doi: 10.3389/fvets.2021.636715 (PMC7952442; doi:10.3389/fvets.2021.636715)
Supplement: Supplementary file 1 [file Data_Sheet_1.docx]

**Table S1.** *E. coli* Primers and reference strains used in PCR reactions

| **Pathogen** | **Name of gene** | **Primer sequence (5’- 3’)** | **Reference strain** | **References** |
| --- | --- | --- | --- | --- |
| EPEC/EHEC | *eaeA* | (F) ATGCTTAGTGCTGGTTTAGG | DSM8695 | Stanilova et al. (2011) |
|  |  | (R) GCCTTCATCATTTCGCTTTC |  |  |
| EAEC | *éagg* | (F) AGACTCTGGCGAAAGACTGTATC | DSM10974 | Pass and Odedra (2004) |
|  |  | (R) ATGGCTGTCTAATAGATGAGAAC |  |  |
| EIEC | *ípaH* | (F) GTTCCTTGACCGCCTTTCCGATACCGTC | DSM9025 | Vidal et al. (2005) |
|  |  | (R) GCCGGTCAGCCACCCTCTGAGAGTAC |  |  |
| EHEC | *fliCH7* | (F) TACCATCGCAAAAGCAACTCC | O157H7 | Cebula et al. (1995) |
|  |  | (R) GTCGGCAACGTTAGTGATACC |  |  |
|  | *sxt* | (F) GAGCGAAATAATTTATATGTG |  |  |
|  |  | (R) TGATGATGGCAATTCAGTAT |  |  |
| ETEC | *St* | (F) TTTCCCCTCTTTTAGTCAGTCAACTG | DSM10973 | Stacy-Phipps et al. (1995) |
|  |  | (R) GGCAGGATTACAACAAAGTTCACA |  |  |
|  | *Lt* | (F) TGCTATGTGCATACGGAGC |  |  |
|  |  | (R) CCATACTGATTGCCGCAAT |  |  |

**Key**: F – Forward primer; R – Reverse primer

**Table S2**: Genome and assembly characteristics of sequenced *E. coli* isolates from a wastewater treatment plant and its associated waters.

| **Isolate ID** | **Source** | **Accession no.** | **Pathotype** | **Antigen**  **Somatic O)** | **Antigen**  **Flagellar (H)** | **Sequence length (bp)** | **No. of contigs** | **GC content (%)** | **Longest contig size (bp)** | **N50 value (bp)** | **L50 value** |
| --- | --- | --- | --- | --- | --- | --- | --- | --- | --- | --- | --- |
| D18/5 | Downstream | SAMN14219475 | EAEC | - | H31 | 4900408 | 86 | 50.4 | 636758 | 164349 | 8 |
| D47/7 | Downstream | SAMN14219476 | EHEC | O15 | H1 | 6112985 | 443 | 51.2 | 342597 | 98305 | 19 |
| D64/7 | Downstream | SAMN14219478 | EPEC | O42 | H21 | 4822859 | 70 | 50.7 | 509836 | 217577 | 7 |
| D69/7 | Downstream | SAMN14218479 | EAEC | O103 | H43 | 5002862 | 134 | 50.5 | 449873 | 165481 | 10 |
| D77/8 | Downstream | SAMN14219480 | EAEC | O175 | H28 | 5130394 | 69 | 50.6 | 724329 | 187963 | 9 |
| D96/9 | Downstream | SAMN14219481 | EIEC | O15 | H6 | 5321899 | 216 | 50.8 | 683241 | 249887 | 7 |
| D106/9 | Downstream | SAMN14219482 | EAEC | O158 | H26 | 5139637 | 53 | 50.4 | 700234 | 466750 | 5 |
| E13/5 | Effluent | SAMN14219483 | EAEC | O134 | H31 | 4903557 | 43 | 50.7 | 567756 | 313135 | 6 |
| U40/6 | Upstream | SAMN14219485 | EAEC | O15 | H18 | 5091294 | 72 | 50.6 | 457405 | 294914 | 7 |
| U69/7 | Upstream | SAMN14219486 | EIEC | O9 | H9 | 5154438 | 180 | 50.9 | 421954 | 152192 | 10 |
| U88/8 | Upstream | SAMN14219487 | EIEC | O107 | H27 | 4715434 | 131 | 50.8 | 247421 | 113945 | 14 |
| U117/10 | Upstream | SAMN14219488 | EAEC | O17 | H18 | 5221995 | 84 | 50.7 | 545274 | 173673 | 10 |

**Key**: N50 = smallest contig of the size-sorted contigs that make up at least 50% of the respective assembly; L50 = number of contigs that make up at least 50% of the respective total assembly length; - absent

**Table S3**: Distribution of Insertion sequences and intact prophages among *E. coli* isolates

| **Isolate ID** | **MLST** | **Insertion sequences** | **Intact prophages** |
| --- | --- | --- | --- |
| D18/5 | ST372 | ISWpi16, ISCysp11,  ISCpe2, ISDde2, | Entero_fAA91_ss, Escher_vB_EcoM_Eco1230_10 |
| D47/7 | ST69 | MITESen1,  TnEc1,  ISDge12,  ISRtr2 | Entero_fAA91_ss,  Entero_mEp460,  Salmon_118970_Sa13,  Entero_PsP3 |
| D64/7 | ST101 | ISSm4, ISAzsp1,  IS1086, ISAeme6 | Shigel_sfII,  Entero_P2 |
| D69/7 | ST218 | ISDha1, ISCfr26,  ISBast1, ISChy6 | Salmon_Fels_2,  Entero_P88 |
| D77/8 | ST200 | ISEc43,  ISEc47,  ISEc23,  IS682 | Psuedo_phiSA1,  Escher_D108,  Entero_DE3,  Entero_fAA91_ss |
| D96/9 | ST69 | ISCs6, TnAs2, ISAlw35, ISNm13 | Entero_mEp460 |
| D106/9 | **ST11351** | ISBmu2, ISCbe4,  ISBm3,  ISMno20 | Salmon_SSU5,  Escher_pro147,  Entero_lambda |
| E13/5 | ST569 | ISEc42,  MITEEc1,  ISIN,  ISEc30, | Salmon_Fels_2,  Entero_PsP3,  Yersin_L_413C,  Entero_sf101,  Shigel_SfII |
| U40/6 | ST69 | IS609, ISEc44, ISSen6, ISAen6 | Entero_mEp460 |
| U69/7 | ST10 | MITEEc1, IS621, MITEype1, ISCro3 | Entero_PsP3 |
| U88/8 | ST10 | IS621, ISPa94,  ISDeac1,  ISSty2 | Entero_mEp237,  Salmon_Fels_2,  Shigel_SfII |
| U117/10 | ST69 | ISEc46, ISSba14,  ISAeme8,  MITEEc1 | Entero_mEp460,  Shigel_SfII, |

**Fig. S1:**  Distribution and incidence of virulence genes per *E. coli* isolate.
